# Expression of *N*-Acetylgalactosamine 4-Sulfate 6-*O*-Sulfotransferase Involved in Chondroitin Sulfate Synthesis Is Responsible for Pulmonary Metastasis

**DOI:** 10.1155/2013/656319

**Published:** 2013-02-20

**Authors:** Shuji Mizumoto, Moto Watanabe, Shuhei Yamada, Kazuyuki Sugahara

**Affiliations:** ^1^Laboratory of Proteoglycan Signaling and Therapeutics, Frontier Research Center for Post-Genomic Science and Technology, Graduate School of Life Science Hokkaido University, West-11, North-21, Kita-ku, Sapporo, Hokkaido 001-0021, Japan; ^2^Department of Pathobiochemistry, Faculty of Pharmacy, Meijo University, 150 Yagotoyama, Tempaku-ku, Nagoya 468-8503, Japan

## Abstract

Chondroitin sulfate (CS) containing E-disaccharide units, glucuronic acid-*N*-acetylgalactosamine(4, 6-*O*-disulfate), at surfaces of tumor cells plays a key role in tumor metastasis. However, the molecular mechanism of the metastasis involving the CS chain-containing E-units is not fully understood. In this study, to clarify the role of E-units in the metastasis and to search for potential molecular targets for anticancer drugs, the isolation and characterization of Lewis lung carcinoma (LLC) cells stably downregulated by the knockdown for the gene encoding *N*-acetylgalactosamine 4-*O*-sulfate 6-*O*-sulfotransferase (GalNAc4S-6ST), which is responsible for the formation of E-units in CS chains, were performed. Knockdown of *GalNAc4S-6ST* in LLC cells resulted in a reduction in the proportion of E-units, in adhesiveness to extracellular matrix adhesion molecules and in proliferation *in vitro*. Furthermore, the stable downregulation of *GalNAc4S-6ST* expression in LLC cells markedly inhibited the colonization of the lungs by inoculated LLC cells and invasive capacity of LLC cells. These results provide clear evidence that CS chain-containing E-units and/or GalNAc4S-6ST play a crucial role in pulmonary metastasis at least through the increased adhesion and the invasive capacity of LLC cells and also provides insights into future drug targets for anticancer treatment.

## 1. Introduction 

 Chondroitin sulfate (CS) is a sulfated glycosaminoglycan (GAG) that covalently attaches to core proteins to form CS-proteoglycans (CS-PGs) [1, 2]. CS/DS-PGs are ubiquitous in extracellular matrices (ECMs) and at cell surfaces in various tissues and regulate various physiological events such as cell proliferation, cytokinesis, morphogenesis, and viral infections through interaction with various proteins [3, 4]. Furthermore, CS-PGs at the tumor cell surface and in the ECM are related to metastatic potential and facilitate tumor invasion by enhancing integrin-mediated cell adhesion, motility, and intracellular signaling [5–8]. Interestingly, the binding of P-selectin to a tumor cell surface depends on the expression of the *CHST11* gene encoding chondroitin 4-*O*-sulfotransferae-1 (C4ST-1), and CSPG4 (also known as melanoma-associated CSPG) serves as a P-selectin ligand through its CS side chains and participates in the binding of P-selectin to highly metastatic breast cancer cells [9]. Further, the expression of a PG, versican, is upregulated in various types of tumors including lung cancer, as a macrophage activator that acts through Toll-like receptor-2 and its co-receptors Toll-like receptor-6 and CD14 [10]. 

 The sugar backbone of CS chains is a linear polysaccharide consisting of repeating disaccharide units, [–4GlcUA*β*1-3GalNAc*β*1–]_*n*_, where GlcUA and GalNAc represent D-glucuronic acid and *N*-acetyl-D-galactosamine, respectively, [4]. CS chains are modified by specific sulfotransferases, which catalyze the transfer of a sulfate group from 3′-phosphoadenosine 5′-phosphosulfate to C-2 of GlcUA and C-4 and/or C-6 of GalNAc. The combination of sulfated positions results in several disaccharide units with different sulfation patterns and yields enormous structural diversity in terms of sequence  [3, 11]. For instance, monosulfated GlcUA-GalNAc(4-*O*-sulfate) and GlcUA-GalNAc(6-*O*-sulfate) disaccharides, abbreviated as A- and C-units, are involved in the differentiation of chondrocytes and the development of a spine, respectively [12–15]. Representative disulfated disaccharide units are GlcUA(2-*O*-sulfate)-GalNAc(6-*O*-sulfate) (D-units) and GlcUA-GalNAc(4-*O*-, 6-*O*-disulfate) (E-units), which function in neuritogenesis [16–18] and viral infections [19, 20], respectively. 

 Recently, it has been reported that the expression of E-unit-containing structures recognized by an anti-CS-E phage display antibody, GD3G7 [21, 22], is increased in ovarian and pancreatic cancer tissues, resulting in alterations in tumor growth and tumor cell motility through the regulation of the signaling of vascular endothelial growth factor (VEGF) and the cleavage of CD44, respectively [22, 23]. Further, the expression of GalNAc 4-*O*-sulfate 6-*O*-sulfotransferase (GalNAc4S-6ST), which transfers a sulfate group to position 6 of GalNAc(4-*O*-sulfate) in A-disaccharide units formed by C4ST and is responsible for the formation of E-units [24, 25], is increased in colorectal cancer tissues compared with paired normal mucosa [26]. Thus, these observations appear to suggest that E-units in CS chains are upregulated in tumor tissues compared to normal tissues.

 Moreover, we demonstrated that the expression of *GalNAc4S-6ST * and the proportion of disulfated E-disaccharides are increased in highly metastatic compared to low metastatic Lewis lung carcinoma (LLC) cells [27]. The colonization by intravenously injected LLC cells of mouse lungs was efficiently inhibited by preinjected CS-E polysaccharides, rich in E-units, derived from squid cartilage and by the anti-CS-E phage display antibody, GD3G7 [27], suggesting *GalNAc4S-6ST* and/or E-unit-containing CS chains to be involved in the pulmonary metastasis of LLC cells. In addition, the GAG-binding receptor in mouse lung was recently identified as Receptor for Advanced Glycation Endproducts (RAGE), which showed high affinity toward CS-E and heparan sulfate chains [28]. However, the exact structural features of GAGs remain to be investigated especially because RAGE could interact with both CS-E and heparan sulfate with high affinity [28]. In the present study, to clarify the role of E-units in metastasis, the isolation and characterization of LLC cells stably downregulated for the gene encoding GalNAc4S-6ST by knockdown using short hairpin RNA were performed.

## 2. Materials and Methods

### 2.1. Materials

The following sugars and enzymes were purchased from Seikagaku Biobusiness Corp. (Tokyo, Japan): CS-A from whale cartilages; CS-E from squid cartilages; six unsaturated standard disaccharides derived from CS; chondroitinase ABC (EC 4.2.2.20) from *Proteus vulgaris*; chondroitinase AC-II (EC 4.2.2.5) from *Arthrobacter aurescens*. Short hairpin RNA- (shRNA-) expressing plasmids (cat no.: sc-145317-SH) specific to mouse GalNAc4S-6ST, which target 5′-CUACAAUGUGGGAUAACAA-3′, 5′-CAAGACACCCUUAGAAUGU-3′, and 5′-GAACACUCGUGCUUAUACU-3′ and scrambled nucleotide sequence-containing control-shRNA plasmids (cat no.: sc-108060), shRNA transfection reagent, and shRNA plasmid transfection medium were purchased from Santa Cruz Biotechnologies, Inc (Santa Cruz, CA, USA). 

### 2.2. Animals and Cell Lines

Seven-week-old male C57BL/6J mice and LLC cells were obtained from Japan SLC (Hamamatsu, Japan) and RIKEN Cell Bank (Tsukuba, Japan), respectively. All the experiments were performed under the experimental protocol approved by the local animal care committee of Hokkaido University. 

### 2.3. Isolation of LLC Clones Stably Downregulated for the Expression of *GalNAc4S-6ST*

The *GalNAc4S-6ST (Chst15)* and *control shRNA* plasmids were individually transfected according to the manufacturer's instructions. The resultant puromycin-resistant colonies were subcultured on a 96-well culture plate by limiting dilution at a low density (1 cell/well), and were propagated.

### 2.4. Quantitative Real-Time PCR

Total RNA was extracted from each clone using an RNA isolation kit, illustra RNAspin Midi (GE Healthcare, Buckinghamshire, UK). Each cDNA was synthesized from ~1 *μ*g of the total RNA using Moloney murine leukemia virus-reverse transcriptase (Promega, Madison, WI, USA) and an oligo(dT)_16_ primer (Hokkaido System Science, Sapporo, Japan). The primer sequences used were as follows: for *GalNAc4S-6ST *(153 bp), 5′-TATGACAACAGCACAGACGG-3′ (forward) and 5′-TGCAGATTTATTGGAACTTGCGAA-3′ (reverse); for *glyceraldehyde-3-phosphate dehydrogenase* (*G3pdh*) (205 bp), 5′-CATCTGAGGGCCCACTG-3′ and 5′-GAGGCCATGTAGGCCATGA-3′. Quantitative real-time PCR was performed using a Brilliant II SYBER Green QPCR master mix in Mx3005P real-time QPCR (Agilent Technologies, Santa Clara, CA, USA). The level of *GalNAc4S-6ST* mRNA was normalized to that of the transcript of *G3pdh*.

### 2.5. Analysis of Disaccharide Composition of CS/DS Chains Isolated from LLC Cells

To obtain evidence that the knockdown of *GalNAc4S-6ST* results in a reduction in E-units [GlcUA-GalNAc(4-*O*-, 6-*O*-disulfates)] in CS chains from LLC cells, the CS disaccharide composition of each clone was determined as described previously [29, 30]. 

### 2.6. Assays for Lung Metastasis

To investigate the effects of the knockdown of *GalNAc4S-6ST* on experimental tumor metastasis, the control shRNA- and GalNAc4S-6ST-shRNA/LLC cells (1 × 10^6^ cells/mouse) were injected into a lateral tail vein of C57BL/6 mice as described in [27]. Three weeks after the injection, the animals were sacrificed, and the number of visible and parietal nodules in the lung was counted by two observers in a blinded fashion. 

### 2.7. Cell Adhesion Assay

Plastic cover slips (10 × 10 mm) were precoated with 10 *μ*g/mL of laminin (Invitrogen), fibronectin, or type IV collagen (BD Biosciences, San Jose, CA, USA) overnight at 4°C and then washed with phosphate-buffered saline twice. The control shRNA and GalNAc4S-6ST-shRNA/LLC cells were seeded on cover slips in 24-well plates at 5 × 10^4^ cells/mL in serum-free Dulbecco's modified Eagle's medium (DMEM) and incubated for 1~2 h at 37°C. The supernatant with nonadherent cells was removed by three washes with a warmed culture medium. Attached cells were fixed, stained with the Diff-Quik staining kit (Sysmex International Reagents Co., Kobe, Japan), and counted in an area of 2 mm^2^.

### 2.8. Cell Migration and Invasion Assays

The ability of GalNAc4S-6ST-shRNA/LLC cells to invade and migrate was assessed using the BD BioCoat chamber with or without Matrigel (BD Biosciences) *in vitro*, respectively. The lower chambers were filled with DMEM containing 10% FBS, and single cell suspensions of LLC cells (2 × 10^4^ cells/500 *μ*L) in serum-free DMEM were placed in the upper chamber. After incubation for 26 h, the cells, which migrated or invaded through the membrane alone or the Matrigel-coated membrane, respectively, and remained bound to the underside of the membranes, were stained with the Diff-Quik and counted in five random microscopic fields/filters.

### 2.9. Cell Proliferation Assay

The control shRNA and GalNAc4S-6ST-shRNA/LLC cells were seeded in 96-well plates at 2,000 cells/well in DMEM containing 10% FBS and cultured for various periods. The number of living cells was measured at each time point using TetraColor One (Seikagaku Biobusiness Co.) according to the manufacturer's instructions. Triplicate cultures were used for each sample. After incubation for 1 h at 37°C, the developed color was measured at 450 nm using a microplate reader (Bio-Rad, Hercules, CA, USA). 

## 3. Results

### 3.1. Isolation and Characterization of the LLC Cells Expressing shRNAs Specific for *GalNAc4S-6ST*

**Figure 1:**
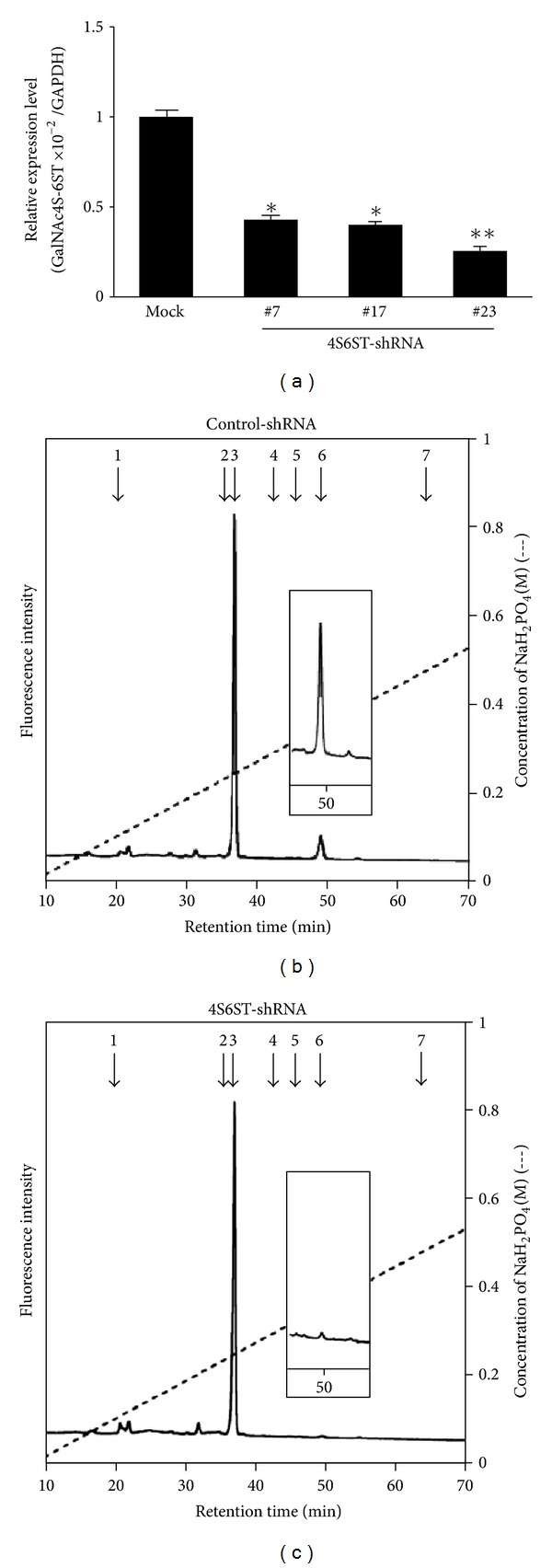
Quantitative real-time PCR analysis of the *GalNAc4S-6ST* transcript and profile of sulfation pattern of CS in the LLC-4S6ST-shRNA cells. (a) Total RNA was extracted from the control- (mock) or GalNAc4S-6ST-shRNA/LLC cells, and cDNA was synthesized by reverse transcriptase. Real-time PCR was conducted using the cDNAs, *Taq* polymerase, and SYBER Green. The expression of *GalNAc4S-6ST* was individually normalized to that of *G3pdh*. The assay was performed at least twice in triplicate, and representative results are shown. Values represent the mean ± SD. *P < 0.005; **P < 0.001 versus control by one-way ANOVA with Dunnett's adjustment. (b, c) Anion-exchange HPLC of disaccharides obtained from the digests of CS derived from control- and GalNAc4S-6ST-shRNA/LLC cells with a mixture of chondroitinases ABC and AC-II. The GAG-peptide preparations from LLC cells, which stably express control-shRNA (b) or *4S6ST-shRNA* (c), were individually digested with a mixture of chondroitinases ABC and AC-II. Each digest was labeled with a fluorophore 2AB as detailed in Section 2 and analyzed by anion-exchange HPLC on an amine-bound silica PA03 column using a linear gradient of NaH_2_PO_4_ as indicated by the dashed lines. The eluate was monitored by fluorescence intensity with the excitation and emission wavelengths of 330 and 420 nm, respectively. The insets show magnified chromatograms (10-fold) around the elution position of ΔE units. The positions of the 2AB-derivatized authentic disaccharides are indicated by numbered arrows: 1: ΔO, ΔHexUA-GalNAc; 2: ΔC, ΔHexUA-GalNAc(6-*O*-sulfate); 3: ΔA, ΔHexUA-GalNAc(4-*O*-sulfate); 4: ΔD, ΔHexUA(2-*O*-sulfate)-GalNAc(6-*O*-sulfate); 5: ΔB, ΔHexUA(2-*O*-sulfate)-GalNAc(4-*O*-sulfate); 6: ΔE, ΔHexUA-GalNAc(4-*O*-, 6-*O*-disulfate); 7: ΔT, ΔHexUA(2-*O*-sulfate)-GalNAc(4-*O*-, 6-*O*-disulfate).

To isolate the clones of LLC cells in which the *GalNAc4S-6ST *gene was suppressed, the vectors expressing shRNAs, which also contain the puromycin-resistance gene, specific for mouse *GalNAc4ST-6ST* were introduced into the cells. Fifty-seven LLC clones resistant to puromycin were isolated (LLC-4S6ST-shRNA). To examine the efficacy of the knockdown of *GalNAc4S-6ST* by specific shRNA, quantitative real-time PCR was conducted after the extraction of total RNA from ten randomly selected clones, followed by the synthesis of the cDNA. The isolated LLC-4S6ST-shRNA clones (nos. 7, 17, and 23) showed the downregulation of *GalNAc4S-6ST* (30~40% of the control-shRNA clones) (Figure 1(a)). Thus, these three clones were utilized for further analyses. 

 To further characterize the effects of the knockdown of the *GalNAc4S-6ST* gene on the amount of E-units, the disaccharide composition of CS chains, which were prepared from each clone as a GAG-peptide fraction, was determined. Representative chromatograms are shown in Figures 1(b) and 1(c), and the composition and amounts of the disaccharides are summarized in Table 1. The data obtained from the digest of the GAG-peptides using a mixture of chondroitinases ABC and AC-II revealed that the low sulfated disaccharide, HexUA-GalNAc(4-*O*-sulfate) (A), where HexUA represents hexuronic acid (*β*-GlcUA or *α*-iduronic acid), was a major disaccharide unit, ~94% (Table 1), and HexUA-GalNAc(4-, 6-*O*-disulfate) (E) accounted for ~6% of all the disaccharides in the LLC-control-shRNA cells consistent with a previous report [28]. On the other hand, a drastic reduction in the proportion of E-units, to 0.5~1.9%, was observed in the LLC-4S6ST-shRNA clones (nos. 7, 17, and 23) compared to the LLC-control-shRNA cells (Table 1). Thus, these clones were utilized to further experiments. It should be noted that the amounts of total disaccharides recovered were less in GalNAc4S-6ST-shRNA clones (213, 323, and 246 pmol/mg acetone powder) compared to wild-type cells and control-shRNA clones (754, 1348, 924, and 534 pmol/mg acetone powder), suggesting that GalNAc4S-6ST or CS chains containing E-units may affect the amounts or lengths of CS chains, or other CS-biosynthetic enzymes. In contrast, bone marrow-derived mast cells from the knockout mice of GalNAc4S-6ST synthesized larger CS chains than the wild type, and levels of the chondroitin 4-*O*-sulfotransferase-1 and chondroitin 6-*O*-sulfotransferase-1 transcripts in the homozygous mutant mice were higher than those in the wild type [25]. Thus, the contrasting effect on the biosynthesis of CS by the expression of GalNAc4S-6ST in LLC cells remains to be elucidated.

### 3.2. Effects of the Knockdown of the *GalNAc4S-6ST* Gene in LLC Cells on Pulmonary Metastasis

**Figure 2:**
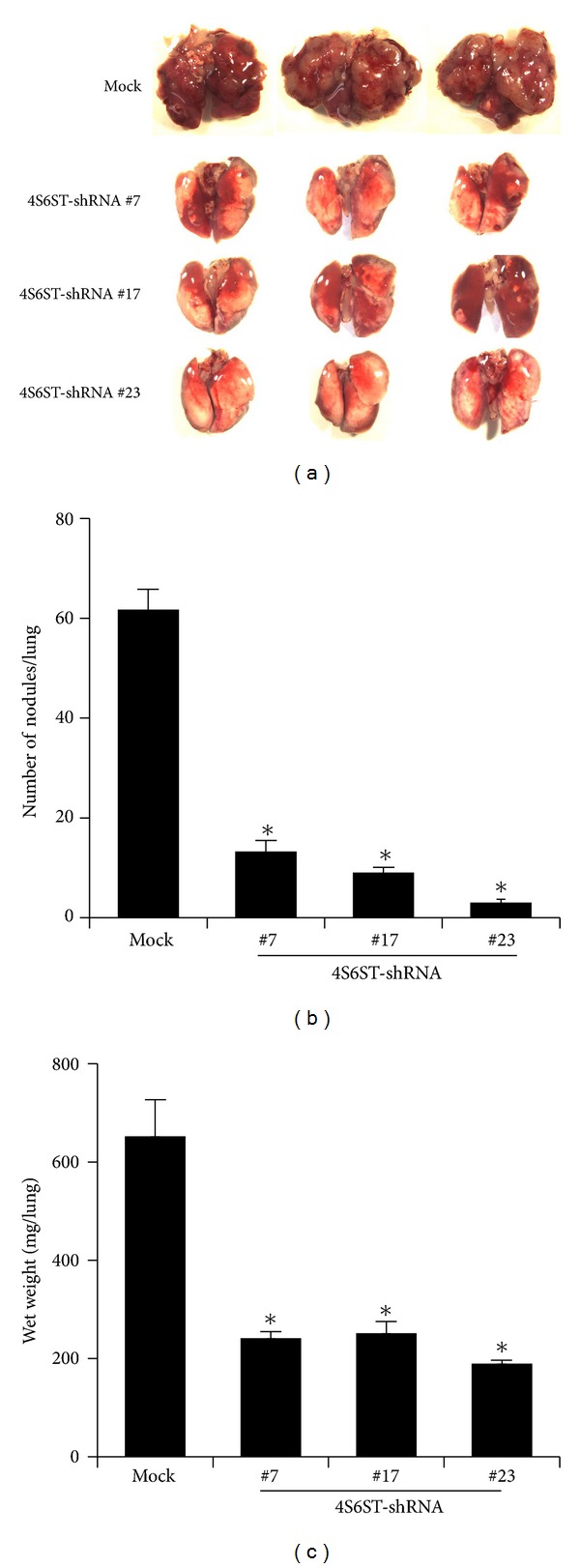
Effects of the knockdown of *GalNAc4S-6ST* on the pulmonary metastasis of LLC cells. Control- or GalNAc4S-6ST-shRNA/LLC-cell suspensions of 1 × 10^6^ cells in 200 *μ*L of DMEM were injected into a tail vein of C57BL/6J mice, and 21 days later, the number of tumor foci in the lungs was recorded. Six mice were used per group. Representative lungs from mice injected with the LLC cells stably expressing control- (mock) or *GalNAc4S-6ST-shRNA * (*4S6ST-shRNA*) are shown in (a). The number of colonies (b) and the wet weight (c) of the lungs from mice injected with each LLC clone were measured. Data represent the mean ± S.D. for two independent experiments. *P < 0.001 versus control by one-way ANOVA with Dunnett's adjustment.

To assess the influence of the knockdown of the *GalNAc4S-6ST* gene and the resulting reduction of E-units in LLC cells on pulmonary metastasis, the LLC-4S6ST-shRNA clones were individually inoculated into mice via a tail vein. Three weeks later, the mice were sacrificed, and pulmonary metastasis was evaluated by counting tumor foci on the lung surface and weighing the lung tissues. As expected, the knockdown of *GalNAc4S-6ST* drastically reduced the metastasis of LLC cells compared with that in mice injected with the LLC-control-shRNA (Figure 2), suggesting a crucial role for the cell surface CS chains containing E-units in the pulmonary metastasis of LLC cells. 

### 3.3. Characterization of the LLC-4S6ST-shRNA Cells *In Vitro*

**Figure 3:**
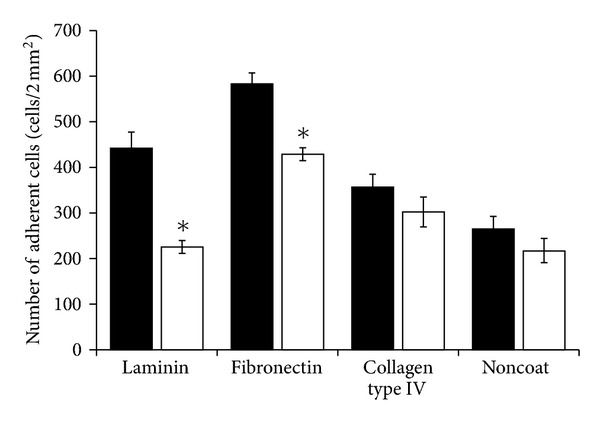
Effects of the knockdown of *GalNAc4S-6ST* on the adhesion of LLC cells. LLC cells stably expressing control-shRNA (filled bars) or *GalNAc4S-6ST-shRNA* (open bars) were seeded on laminin-, fibronectin-, or collagen type IV-precoated plastic cover slips for 1-2 h. The cells were stained with Diff-Quik, and the adherent cells were counted. Average values obtained from three clones of both control-shRNA (nos. 5, 10, and 14) and GalNAc4S-6ST-shRNA (nos. 7, 17, and 23) are shown, and the experiments were performed in triplicate. Error bars indicate ±S.D. of triplicate samples. *P < 0.05 versus control-shRNA by Student's *t*-test.

LLC cells are frequently utilized as a model for experimental lung metastasis [31]. After their inoculation into the tail of mice, LLC cells reach the lung and may bind to the surface of the vascular endothelium through adhesion to ECM molecules [32–34]. Hence, to assess the change in the adhesiveness of LLC cells by the knockdown of *GalNAc4S-6ST*, the adhesive capacity of the LLC-4S6ST-shRNA was examined using adhesion molecules in ECM including laminin, fibronectin, and type IV collagen. The number of LLC-4S6ST-shRNA cells adhering to laminin or fibronectin but not to type IV collagen was significantly reduced (51 and 73% of the control-shRNA expressing LLC cells, resp.) (Figure 3). These observations indicate that CS chains at the LLC cell surface containing E-units may be involved in the initial cell adhesion to ECM molecules produced on the vascular endothelium in the lung during metastasis. 

**Figure 4:**
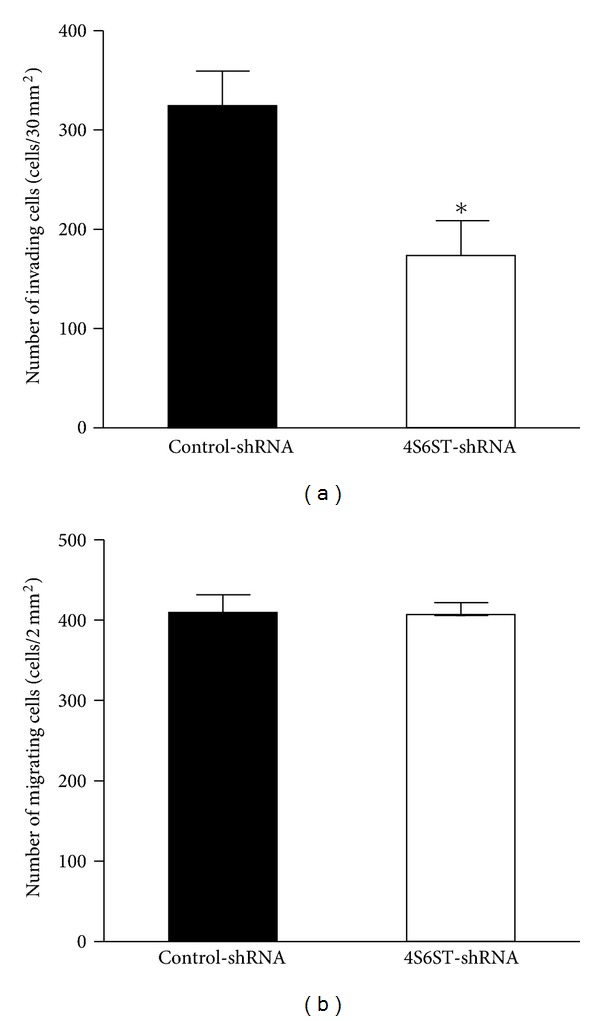
Effects of the knockdown of *GalNAc4S-6ST* on the invasion and migration of LLC cells. LLC cells stably expressing control-shRNA (filled bars) or *GalNAc4S-6ST-shRNA* (open bars) were plated on BD BioCoat chambers in the absence of fetal bovine serum. Cell invasion and migration were measured with or without Matrigel (BD Biosciences), respectively, as described in Section 2. Effects of the knockdown of *GalNAc4ST-6ST* on the invasion (a) and migration (b) of the LLC cells are summarized. The data represent the mean values ± S.D. for three clones of both control-shRNA (nos. 5, 10, and 14) and GalNAc4S-6ST-shRNA (nos. 7, 17, and 23). Three independent experiments were performed, and the representative results are shown. *P < 0.05 versus control-shRNA by Student's *t*-test.

 Along with the adhesion of LLC cells to a target tissue or cell, cell migration and invasion are also important to cancer progression and metastasis [35]. Next, to determine if the inhibition of the enzyme GalNAc4S-6ST using shRNA affects the migration and invasion of LLC cells; the invasive or migratory potential of LLC-4S6ST-shRNA cells was examined *in vitro* using a Boyden chamber coated with or without Matrigel, respectively. Although the migration was not significantly diminished by the knockdown of *GalNAc4S-6ST* when compared with that using a control-shRNA (Figure 4(b)), down-regulation of E-units in CS chains caused by the knockdown of *GalNAc4S-6ST* resulted in a significant decrease in the invasion of LLC-4S6ST-shRNA cells through Matrigel as compared with the control knockdown (Figure 4(a)), indicating that *GalNAc4ST-6ST* and/or the E-units in CS chains regulate invasion but not migration during the metastasis of LLC cells, being consistent with a previous report [27].

**Figure 5:**
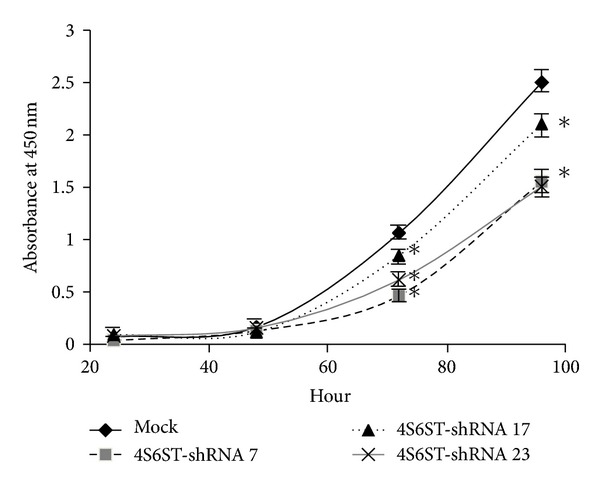
Effects of the knockdown of *GalNAc4S-6ST* on the proliferation of LLC cells. LLC cells stably expressing control-shRNA (mock) or *GalNAc4S-6ST-shRNA* (*4S6ST-shRNA*) were seeded on 96-well plates at 2 × 10^3^ cells/well and incubated at 37°C for the assessed period. Cell numbers were measured every 24 h using TetraColor One reagent containing a tetrazolium and electronic carrier as described in Section 2. The data represent the mean ± S.D. for three clones of both control-shRNA (nos. 5, 10, and 14) and GalNAc4S-6ST-shRNA (nos. 7, 17, and 23). Three independent experiments were performed, and representative results are shown. *P < 0.001 versus control-shRNA by Student's *t*-test.

 It is possible that LLC-4S6ST-shRNA cells are not so metastatic as we expect because of a possibility that they may grow more slowly than LLC-control-shRNA cells, which also partly contributes the lower metastatic capcity of LLC-4S6ST-shRNA cells. To address this issue, the LLC cells were plated at a low density and the growth rate was determined. LLC-4S6ST-shRNA cells grew more slowly than control cells according to the results of a cell proliferation assay (Figure 5). These results indicate that the suppression of the metastasis of LLC cells by the knockdown of *GalNAc4S-6ST* shown in Figure 1(b) was partly due to a depression of cell growth potential. 

## 4. Discussion

In the present study, to evaluate the involvement of E-units in CS chains expressed at the surface of LLC cells in tumor metastasis, we used an animal model of lung carcinoma and shRNA specific for the *GalNAc4S-6ST *gene, which is responsible for the formation of E-units [24, 25]. Firstly, *GalNAc4S-6ST* was overexpressed in LLC cells using several expression vectors such as pcDNA3.1/myc-His, pEF6/V5-His, and pIRESneo3, although the expression of *GalNAc4S-6ST* and E-units in CS chains was not enhanced for some unkown reason(s) (data not shown). Therefore, we assessed functions of E-units in the experimental metastatic model by knockdown of *GalNAc4S-6ST* using an shRNA-expressing vector in LLC cells. 

CS-E interacts with heparin or heparan sulfate-binding proteins such as fibroblast growth factors, VEGF, pleiotrophin, and midkine [22, 36]. E-unit-containing structures of CS chains at the cell surface are important for the binding of LLC cells to laminin and fibronectin (Figure 3), which also interact with heparan sulfate [37–39]. Thus, E-units in CS chains in addition to heparan sulfate chains at the surface of LLC cells may also contribute the binding to laminin and/or fibronectin on the luminal side of the vascular endothelial cells in lungs. 

Furthermore, endothelial carbohydrate-binding proteins, E- and P-selectins, play a role in the pulmonary metastasis of B16 melanoma cells [40]. It has also been reported that CS-E interacts with the adhesion molecules L- and P-selectins, and that CS-PGs at the surface of the metastatic breast cancer cell line are major P-selectin ligands on the endothelium [41, 42]. Hence, CS chains containing E-units may be involved in the adhesion of LLC cells through such cell adhesion molecules. In fact, most recently, we identified RAGE, which is a member of the immunoglobulin superfamily predominantly expressed in the lung, as a receptor for CS-E involved in pulmonary metastasis [28]. Together, the interaction of cell adhesion molecules or receptors including P-selectin and RAGE expressed on the endothelium at secondary target tissues with CS-containing E-units expressed on malignant cell surfaces play major or some roles in the targeting of tumor cells to lungs. 

It has been demonstrated that matrix metalloproteinase-9 is critical for the invasion and metastasis of LLC cells [43], and that heparan sulfate-proteoglycan, syndecan-2, functions as a suppressor for matrix metalloproteinase-2 activation depending on the heparan sulfate side chains on LLC cells [44]. Moreover, melanoma CS-PG regulates membrane-type 3 matrix metalloproteinase and invasion of melanoma cells [45]. The invasive ability of the LLC-4S6ST-shRNA cells in Matrigel was lower than that of the LLC-control-shRNA cells (Figure 4). Hence, CS-containing E-units as well as heparan sulfate may regulate metalloproteinases at the surfaces of LLC cells.

Metastasis is completed via processes involving growth, survival, and neoangiogenesis [32, 33]. CS-E interacts with various heparin-binding proteins such as fibrobrast growth factors, midkine, and pleiotrophin [36]. Further, VEGF binds to CS-E expressed in tumor blood vessels *in vitro* [22]. Our findings (Figure 5) together with these observations prompted us to speculate that CS chains containing E-units may participate as a regulator of VEGF signaling in the proliferation of tumor cells and tumor angiogenesis. In fact, GAG side chains including heparan sulfate and CS of neuropilin-1, a coreceptor for VEGF that augments angioplastic events through VEGF receptor-2, are critical for the reactivity to VEGF in endothelial cells and smooth muscle cells [46]. 

Interestingly, CS-PG, versican, functions as a macrophage activator that acts through Toll-like receptor-2, resulting in the production of tumor-necrosis factor-*α* and strong enhancement of the metastatic growth of LLC [10]. Notably, versican contains E-units in the CS side chains [42]. Thus, these observations with our results raise the possibility that E-unit-containing structures in the CS side chains of versican may contribute to the growth and/or metastasis of LLC cells. 

## 5. Conclusions 

In the present study, a reduction in the expression of *GalNAc4S-6ST* or the proportion of E-unit-containing CS chains effectively suppresses metastatic lung carcinoma through the reduction in adhesiveness, invasion, and proliferation but not the migration of LLC cells. Recently, we identified RAGE, which is specifically expressed in the normal lung, as a receptor for CS-E involved in the pulmonary metastasis [28]. The siRNA of the *GalNAc4S-6ST* gene and CS-E mimetics including small molecular inhibitors that bind RAGE are potential targets for anticancer therapies.

## Figures and Tables

**Table 1:** Disaccharide composition of CS chains in the control- and 4S6ST-shRNA/LLC cells. The GAG-peptide preparation from each cell line was digested with a mixture of chondroitinases ABC and AC-II and analyzed by anion-exchange HPLC after labeling with a fluorophore 2AB as detailed in Section 2.

	Wild type	GalNAc4S-6ST-shRNA			Control-shRNA		
		No. 7	No. 17	No. 23	No. 5	No. 10	No. 14
	pmol/mg acetone powder (mol%)^b^						

ΔA^a^	675 (93.6)	209 (98.1)	321 (99.4)	243 (98.9)	1,273 (94.4)	867 (93.8)	487 (91.0)
ΔE^a^	46 (6.4)	4 (1.9)	2 (0.5)	3 (1.1)	75 (5.6)	57 (6.2)	48 (9.0)

Total	721 (100)	213 (100)	323 (100)	246 (100)	1,348 (100)	924 (100)	535 (100)

^a^ΔA and ΔE represent ΔHexUA-GalNAc(4-*O*-sulfate), and ΔHexUA-GalNAc(4-*O*-, 6-*O*-disulfate), respectively. No other disaccharide units including ΔO, ΔC, or ΔD were detected (data not shown). ΔO, ΔC, and ΔD stand for ΔHexUA-GalNAc, ΔHexUA-GalNAc(6-*O*-sulfate) and ΔHexUA(2-*O*-sulfate)-GalNAc(6-*O*-disulfate), respectively.

^b^Values are expressed in pmol of disaccharide per mg acetone powder as starting materials from the cells and calculated based on the peak areas of the disaccharides detected by anion-exchange HPLC (Figures 1(b) and 1(c)).

## Abbreviations

2AB: 2-Aminobenzamide
CS: Chondroitin sulfate
GAG: Glycosaminoglycan
GalNAc: *N*-Acetyl-D-galactosamine
GalNAc4S-6ST: *N*-Acetylgalactosamine 4-*O*-sulfate 6-*O*-sulfotransferase
GlcUA: D-Glucuronic acid
LLC: Lewis lung carcinoma
PG: Proteoglycan
shRNA: Short hairpin RNA
VEGF: Vascular endothelial growth factor.
